# Influence of Soluble Fillers in Improving Porosity of Handmade Antibiotic-Impregnated Polymethyl Methacrylate (PMMA) Beads: An in-vitro Study

**DOI:** 10.5704/MOJ.1607.002

**Published:** 2016-07

**Authors:** HN Rasyid, S Soegijoko

**Affiliations:** * Department of Orthopaedics and Traumatology, Faculty of Medicine Universitas Padjadjaran / Dr. Hasan Sadikin General Hospital, Bandung, Indonesia; ** Biomedical Engineering Program, School of Electrical Engineering and Informatics, Institut Teknologi Bandung, Indonesia

**Keywords:** Drug delivery system, gentamicin, PMMA, soluble fillers, glycine, sodium chloride

## Abstract

There have been many investigations on non-biodegradable materials acting as an antibiotic carrier for local drug delivery systems based on polymethyl methacrylate (PMMA) beads. However, the material is non-degradable and non-porous so that less than 5% of the encapsulated drug is released. In order to obtain better release of the antibiotics, greater porosity of the beads would be required. Adding fillers could increase the bead’s porosity, thus improving the antibiotic release from the beads. The purpose of the study is to optimize release kinetics of gentamicin from handmade beads by adding fillers such as glycine and sodium chloride in different concentrations. Terms of percolation theory will qualitatively be applied in interpreting the final results. Model beads were made by blending the antibiotics (gentamicin) with powdered PMMA, prepared with the inclusion of glycine and different concentration of sodium chloride in 100% monomer. To determine the gentamicin release, beads were placed in phosphate buffered saline (PBS) and aliquots were taken at designated times to measure the gentamicin concentration. Addition of glycine yielded 16 % release of the total amount of gentamicin incorporated in 24 hours. Subsequent addition of sodium chloride resulted in an increased gentamicin release, with little or no difference in gentamicin release once 16 g or more sodium chloride was added (gentamicin release 100% of the amount incorporated). In conclusion, addition of glycine and sodium chloride resulted in an increased release of gentamicin; however, the combination without sodium chloride seemed to have an inhibitory effect on the gentamicin release.

## Introduction

It is a well established practice in orthopaedic surgery to blend antibiotics into beads of bone cement in the management of musculoskeletal infection. Substantial progress in treating these diseases has been made by the invention of antibiotic-loaded polymethylmethacrylate (PMMA) beads providing the carrier for local delivery of antibiotics in the treatment of infection in orthopaedic practice, such as chronic osteomyelitis^1^.

Studies have been conducted to identify any substances that can increase the porosities of the antibiotic beads but have not yielded optimal results. Therefore, further investigation is needed and sodium chloride, which has a larger particle size than glycine particle size, was chosen as filler and its effect evaluated in creating porosity.

Antibiotic release from bone cement is a complex process and important variables include: type of antibiotic, type of bone cement and the mixing conditions^2-4^. Due to the controlled pharmacokinetics, the antibiotic is released from bone cement beads by way of diffusion, which is dependent on the material properties of the beads.

Release of antibiotics is also strongly influenced by the mixing technique. This study used antibiotic powder and soluble fillers. First, PMMA powder is mixed together, monomer methacrylate is then added, allowed to moisturise and again blended using spatula for two minutes to obtain a free flowing soft paste. This leaves intact as many large crystals as possible to create more porous mixture to increase antibiotic elution rate. The PMMA matrix is structured to provide optimum interaction between the carrier matrix and the antibiotic^4^. Initially however, antibiotics adhering to the surface of the PMMA beads will dissolve rapidly to create a so-called burst-release, which is followed by prolonged release of antibiotics from the matrix by diffusion. Clearly, prolonged release depends on the porosity of bone cement^4^. The effects of porosity and pore size distribution on water permeability and drug release are included in so-called percolation models^5,6^.

Application of the principles of percolation is necessary to design bead systems with an optimal antibiotic release. In essence a “percolating cluster” can be described as a function of relative volume ratios of one or more components in a matrix and when their concentration exceeds a certain threshold, a system with interconnecting pores is obtained yielding optimal antibiotic release^6^.

In order to enhance the porosity of PMMA bone cement, various fillers, such as dextran, glycine, sodium chloride or a second antibiotic have been added^7,8^. Gentamicin is an aminoglycoside and in bead shape constitutes an effective drug delivery system for local antibiotic therapy in bone and soft-tissue infections, and gentamicin concentrations at the site of the infection exceed the minimum inhibitory concentration (MIC) for the infecting organisms. Moreover, MIC values for gentamicin are far lower as resistant bacteria have a MIC level of 4 μg/mL^5,9,10^. The purpose of the study is to optimize the release kinetics of gentamicin from handmade beads by adding glycine and sodium chloride. Results will be qualitatively interpreted according to the terms of percolation theory.

### The Concept of Percolation^9-12^

Some research has been performed to study the effect of pore size, porosities and pore size distribution on water uptake and drug release^5^. Percolation models have been used to characterize the behaviour of porous matrices. The application of the principles of percolation theory is useful to design controlled release porous matrices. “Percolating cluster” can be described as a function of relative volume ratios of one or both components, built by the component particle that are in contact with each other. When the concentration occurs in an infinite ideal system it is known as “percolation threshold” and represents a critical value in each system and can be determined as a critical porosity, therefore, the release kinetics is expected at this point^6,11,12^.

**Fig. 1:**
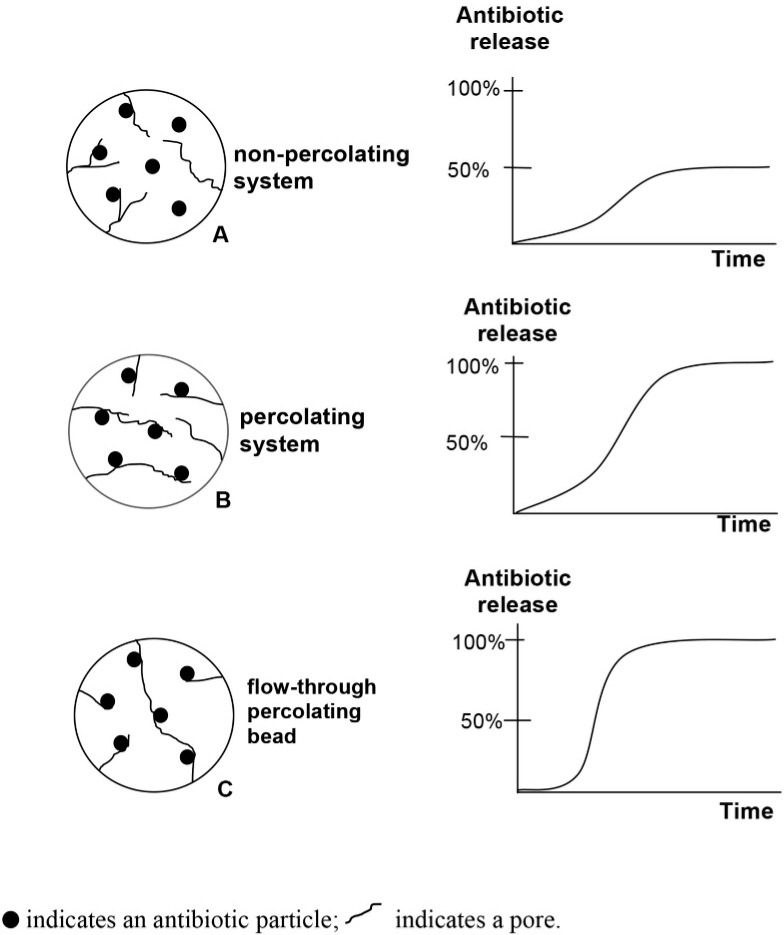
Percolation and hypothetical antibiotic release from porous polymer matrix.

Figure 1 explains the concept of percolation. It represents schematically a bead composed of lattice pores. In Figure 1A, assuming that there are six pores of antibiotics (see holes) and no contact with each other, it can be stated that this condition in non-percolating, the antibiotic will not flow or the antibiotic release only 50% in predicted graph. An antibiotic particle creates a percolating system as long as every drug particle is connected to each other; it means that antibiotics can flow through interconnecting pores. This condition is depicted in Figure 1B. In this system, the antibiotics can be released 100% although it takes time. Figure 1C reveals an interconnection of antibiotic particles, and in this condition, fluid can flow through the percolating system, and the antibiotics are released out to 100%.

**Fig. 2:**
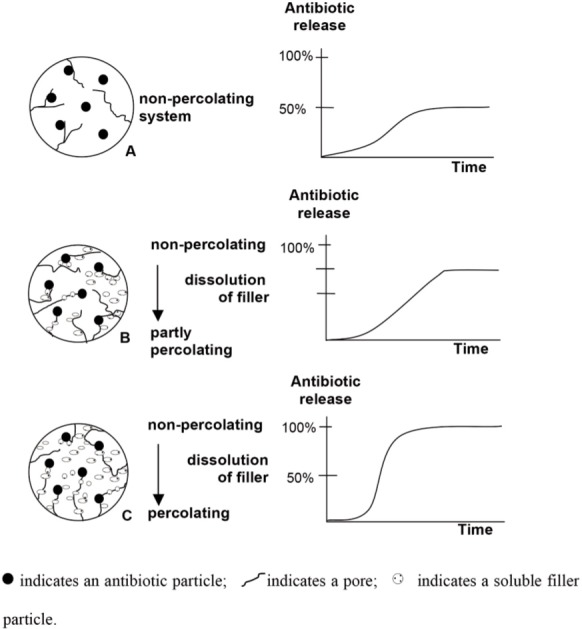
Percolation and hypothetical antibiotic release from a porous polymer matrix with increasing amount of soluble fillers from A (no filler) to C, expressed in terms of the percentage of the total amount of antibiotic incorporated.

Soluble fillers, like glycine, sodium chloride and Kollidon can be used to increase porosities. The soluble filler is a part of percolation systems; therefore, it is able to change the condition from non-percolating to percolating during the solution. After dissolving the filler will cause the antibiotic release, assuming the soluble filler is located around the antibiotic particle (see Figure 2B and C). Glycine or sodium chloride can dissolve very fast, they are incorporated in the bone cement matrix and will be dissolved by water, leaving behind a porous structure. When the content of the soluble filler is high enough (Figure 2C), then, the pores will be interconnected, creating a matrix that is able to allow a continuous release of the antibiotics.

## Materials and Methods

### Particle measurement by laser diffraction

The particle size distributions of gentamicin sulphate, PMMA powder Simplex-P bone cement, crystalline glycineand sodium chloride were measured by laser diffraction spectrometry (Helos H0503, Sympatec GmbH, Germany) using a 200 mm lens. The powders were dispersed at a pressure of 5,00 bar with a Sympatec Rodos dry disperser.

### Bone cement preparation with glycine filler and different concentration of sodium chloride

The mixing was performed as follows: 8 g PMMA powder (Simplex P®, Stryker Howmedica Osteonics, Howmedica, Ireland) was first mixed with 0.2 g of gentamicin sulphate powder (Gracia Pharmaceutical, Indonesia), 0.6 g crystalline glycine (Merck, Darmstadt, Germany) and different amounts (0 g, 12 g, 16 g, 20 g, and 24 g) of sodium chloride (Merck, Darmstadt, Germany) in a ceramic bowl for two minutes. Once the antibiotic powder and soluble fillers were fully blended, the monomer (methacrylate/MMA) was poured over the powder, allowed to become soaked and again blended according to the manufacturer instructions using a spatula for two minutes to yield a free flowing paste. Subsequently, beads with an approximate diameter of 12 mm were hand-rolled, for which 30 min were available until complete polymerization.

### Determination of the antibiotic concentration

The beads were placed in 10 ml of PBS and incubated at 37^0^C. At designated time intervals (6, 24, 48, 72, 168, 336 h), 0.5 µL aliquots of the gentamicin-PBS solution were taken and their gentamicin concentrations were measured using an o-phtaldialdehyde reagent, made and stored for 24 hours in a dark environment. The gentamicin sample, ophtaldialdehyde and 2-mercapto ethanol were mixed in equal proportions and stored for 30 minutes at room temperature. The o-phtaldialdehyde reacted with the gentamicin and a chromophoric product was obtained. The absorbance was measured at 332 nm^13^ using a Spectronic 20 Genesys spectrophotometer. (Spectronic Instruments, Inc. Rochester, NY 14625 United States). The percentages gentamicin released out of the total amount incorporated were calculated for all cements used.

## Result

### Particle size measurements

The size of the gentamicin particles used for the *in vitro* study was measured. As can be seen, the 50% distribution of the gentamicin particles is 18.1 µm and PMMA powder is 15.5 µm (Table I).

**Table I:** Particle size distribution of beads components used for beads preparation as measured by laser diffraction. Diameters representing cumulative powder volume up to 10, 50, and 90% respectively (undersize curve)

**Substance**	**d10 [µm]**	**d50 [µm]**	**d90 [µm]**
Gentamicin sulphate	5.9	18.1	42.1
PMMA powder Simplex-P	1.8	15.5	51.6
Crystalline glycine	43.7	271.8	493.1
Sodium chloride	117	304	487.2

It is noted that the soluble filler of sodium chloride particle, 304 µm, had the higher size compared to glycine particles, which were 271.8 µm.

### Gentamicin release after addition of soluble filler

**Fig. 3:**
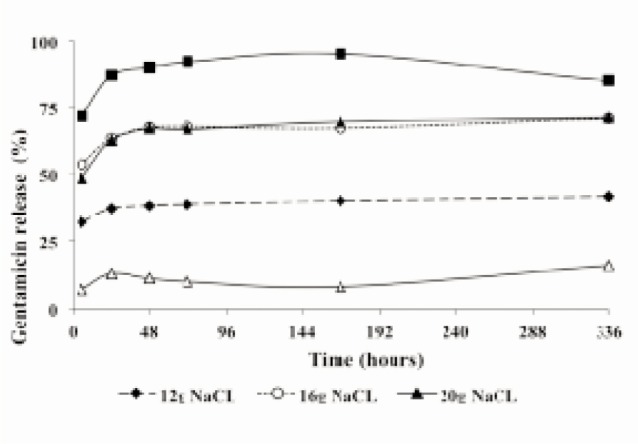
Cumulative gentamicin release (µg/mL) of gentamicin-loaded PMMA in combination with glycine and different concentration of sodium chloride as a function of time during exposure to PBS.

The effects of adding sodium chloride to the gentamicinglycine-PMMA mixture are shown in Figure 3. The addition of sodium chloride clearly enhanced gentamicin release. Optimal release of gentamicin could be seen in the mixture of gentamicin-glycine-sodium chloride, with the highest concentration of sodium chloride (24 g), peak level of the antibiotic release reached 2222.561 µg/mL at 168 h, and the slope continued to decrease and steady (2098.171 µg/mL) until 336 h. The mixture of gentamicin-glycine sodium chloride, peak level reached 331.220 µg/mL at 24 h, and the slope gradually decreased until 168 h (155.610 µg/mL).

## Discussion

Local antibiotic delivery systems have improved the management of complex wounds in musculoskeletal surgery. When a thorough debridement is augmented with high sustained local antibiotic concentration, the progression of bone and soft tissue contamination to become a regional infection may be prevented. Antibiotic-PMMA beads are often used to sterilize and temporarily maintain dead space following debridement surgery.^1,9,10^ The beads are surgically implanted in the debrided bone and covered with soft tissue. Serum, inflammatory fluid, and antibiotic collect in the space (termed a seroma) around the beads. Local antibiotic concentration remained therapeutic for 14 days but became sub-therapeutic at the next measured time point of 28 days. The PMMA beads are left in place for 3 to 4 weeks and then surgically removed^1,9,10^ with consideration for patient safety reasons^14,15^.

Figure 3 shows that the gentamicin release would be increased with an increasing load of sodium chloride or after addition of glycine. Similar doses of glycine increased the release of gentamicin together with increasing doses of sodium chloride; however, glycine without adding more soluble filler such as sodium chloride seemed to have an inhibitory effect on the gentamicin release.

Beads were loaded with the combination of gentamicin and glycine, thus without sodium chloride, showed no enhancement of the gentamicin release at all. In this experiment, addition of sodium chloride was influencing the gentamicin release greatly. For the combination of gentamicin-glycine-sodium chloride in different concentration (12 g, 16 g, and 20 g), gentamicin release could be seen up to 336 h. Remarkably, increasingly higher doses of sodium chloride seemed to have gradually decreased the gentamicin release after peak level at 168 h. It seemed that in this dose, sodium chloride also had an inhibitory effect on the gentamicin release. Sodium chloride has been used to enhance the porosity of acrylic bone cement, and this resulted in an increased released rate of antibiotics in elution studies. The glycine data from this study and the sodium chloride data supported the novel concept of using an inert, inexpensive, soluble filler to increase the elution of antibiotic from PMMA while using a smaller total antibiotic load. By using large amounts of filler such as glycine or sodium chloride, the porosity of handmade beads could be increased to the point that gentamicin would elute in a concentration closer to those seen from commercially prepared beads, and also excessive sodium choride would not cause negative effect in-vivo. Initial rapid release of gentamicin by glycine without added sodium chloride was thousand times lower than gentamicin release with the lowest dosage of sodium chloride. This substance was safe and well tolerated. Moreover, higher dose of antibiotics mixed with monomer did not show good results because the beads would be bulky and would not release the antibiotics.

A combination of glycine and sodium chloride will cause the total available gentamicin to be eluted sooner, shortening the duration of activity. Gentamicin is the most common additive because it has, amongst other features, a good spectrum of concentration-dependent bactericidal activity, thermal stability and high water solubility. Interestingly, when used in combination with gentamicin with the new substance, a synergistic effect appeared.

The effect of adding sodium chloride on the material properties of PMMA has not been studied. A 0.6 g dosage would be expected to compromise the strength and endurance properties of the PMMA.

We conclude that it is possible to optimize the gentamicin release kinetics from handmade PMMA beads by adding glycine and sodium chloride. The gentamicin release is somewhat comparable with the commercial beads, which can be obtained with the addition of glycine and a high dosage of sodium chloride.
